# Associations between feeding patterns and clinical outcomes of Hirschsprung’s disease after surgery: propensity score matching approach

**DOI:** 10.3389/fnut.2025.1553133

**Published:** 2025-07-02

**Authors:** Wei Feng, Xiao Xiang, Xiaohong Die, Jinping Hou, Zhenhua Guo, Wei Liu, Jinfeng Hou, Yi Wang

**Affiliations:** Department of General and Neonatal Surgery, Children’s Hospital of Chongqing Medical University, National Clinical Research Center for Child Health and Disorders, Ministry of Education Key Laboratory of Child Development and Disorders, Chongqing Key Laboratory of Structural Birth Defect and Reconstruction, Chongqing, China

**Keywords:** feeding pattern, clinical outcome, Hirschsprung’s disease, propensity score matching, logistic regression analysis

## Abstract

**Background:**

Feeding pattern is closely related to physical development and health, but the benefit of breast feeding on clinical outcomes of Hirschsprung’s disease (HSCR) remains unknown. This study aimed to investigate the influences of feeding patterns on postoperative outcomes of HSCR using propensity score matching (PSM) analysis.

**Methods:**

The clinical data of 296 patients with HSCR who underwent Laparoscopic-assisted pull-through surgery were retrospectively analyzed. Patients were dichotomized into breast and formula feeding groups. Using propensity score matching (PSM), the two groups were compared for baseline differences and postoperative outcomes. Furthermore, Univariate/multivariate Logistic regression analysis was used to identify feeding pattern as an independent factor of postoperative HAEC and bowel dysfunction.

**Results:**

Of the 296 patients, breast feeding was 73% (216/296). After PSM, patients with formula feeding had higher rates of postoperative undernutrition (risk of malnutrition: 21.05% vs. 8.77%; malnutrition: 28.07% vs. 15.79%, *p* = 0.023), HAEC (47.37% vs. 22.81%, *p* = 0.006), and bowel dysfunction (64.29% vs. 42.11%, *p* = 0.018). Multivariate Logistic regression analysis revealed that formula feeding was an independent risk factor for postoperative HAEC [OR (95% CI) = 6.86 (1.76 ~ 26.79)] and bowel dysfunction [OR (95% CI) = 2.88 (1.06 ~ 7.83)].

**Conclusion:**

Following adjustment for patient characteristics, HSCR patients with breast feeding were associated with lower rates of postoperative undernutrition, HAEC, and bowel dysfunction.

## Introduction

Hirschsprung’s disease (HSCR) is a rare and complex congenital intestinal defect caused by the absence of enteric ganglion cells in the myenteric and submucosal plexuses of the distal intestine, with a prevalence of approximately 1:5000 (1). Surgical resection of the dieased bowel is the primary management for HSCR. In cases with ineffective rectal irrigation, a temporary diverting enterostomy may be necessary (2). Advances in surgical techniques and nursing care have significantly reduced the mortality rate; however, postoperative complications, eg. Hirschsprung-associated enterocolitis (HAEC), bowel dysfunction, and nutritional problems remain concerns (3–5). Therefore, it is important to identify variable conditions that reduce the risk of those postoperative problems.

Feeding patterns significantly influence infant health. Breast milk is the optimal nutrition for infants, with essential nutrients, biologically active ingredients and microbial communities (6). Furthermore, breast feeding introduces the infant to highly diverse and complex bacterial communities, which affects colonization of neonatal gut and maturation of the immune system (7). Compared to formula feeding, breast feeding has shown superior health and developmental outcomes for infants (8). Studies have reported that postoperative HAEC is closely related to gut microbiota imbalance, modulating the gut microbiome by encouraging breast feeding might prevent HAEC progression in HSCR patients (9, 10). Of note, no studies have explored the influences of feeding patterns on postoperative outcomes of HSCR. Understanding this associations between feeding patterns and clinical outcomes in HSCR patients is essential to pediatric surgeons, it helps us to provide rational feeding guidance to promote infant health. Thus, we designed this study to test the hypothesis that breast feeding improves postoperative outcomes in HSCR patients using propensity score matching (PSM) analysis.

## Materials and methods

### Study population

From February 2016 to February 2021, 357 patients who underwent one-stage laparoscopic-assisted pull-through surgery at the Gastrointestinal Neonatal Surgery Department of Children’s Hospital Affiliated with Chongqing Medical University were enrolled in this retrospective study. Inclusion criteria were: (1) HSCR diagnosis confirmed by histopathology, (2) received one-stage Laparoscopic-assisted pull-through surgery (modified Swenson technology by the permanent team, as described in detail in our previous report (11)), (3) cooperation with a follow-up lasting at least three years, and (4) complete medical records. Exclusion criteria included patients with severe malformations affecting HSCR management (7 cases), cognitive disabilities (4 cases), and missing clinical data (17 cases). Patients requiring temporary enterostomy, mainly L-HSCR and TCA cases, were also excluded. It needs to be stated that all patients received standardized follow-up via telephone, internet, or clinic visits.

### Exposure variables

We assessed feeding patterns of these patients through face-to-face interviews with parents or caregivers. Feeding patterns were classified into two types for infants under 6 months of age based on their sources of milk: exclusive breast feeding (only breast milk, including expressed milk) and formula feeding (only formula). Mixed feeding cases (61 patients) were excluded from analysis due to the unknown proportion of breast milk.

### Potential confounders

We retrospectively gathered the following potential confounders based on literature and clinical practice: (1) demographic data (sex, gestational age, birth weight, surgical age, and preoperative comorbidities, and associated malformations); (2) social determinants (residence, relationship of caregivers, educational level of caregivers, and type of insurance); and (3) postoperative findings (pathological type of HSCR and surgical duration).

### Outcome variables

The postoperative outcome variables were length of postoperative hospital stay, postoperative complications within 30 days [graded based on Clavien-Dindo classification system (CCS) (12)], HAEC, nutritional status, defecation function, and health-related quality of life (HRQoL). The definitions or measured criteria for HAEC, nutritional status, defecation function, and HRQoL have been described in our previous reports (11, 13).

### Statistical analysis

Data were analyzed using IBM SPSS version 27.0 and R software (R Foundation for Statistical Computing, Vienna, Austria). Categorical data were expressed by *n* (%) and analyzed using the chi-squared test. The continuous variables were expressed as mean ± standard deviation (SD) and were compared using the independent t test. Propensity score matching (PSM) analysis based on nearest neighbor with a caliper length of 0.25 was performed to reduce potential selection bias with the potential confounders. Quality of match was assessed using the absolute standardized mean difference (SMD) with a goal of ≤ 0.20. This is a valid statistical method to minimize the imbalance in participant characteristics between exposed and unexposed groups by considering the confounding between the two groups (14).

Following matching, univariate/multivariate regression analysis examied the associations between feeding patterns and postoperative HAEC, bowel dysfunction, reporting odds ratios (ORs) with 95% confidence intervals (CIs). A *p*-value < 0.05 was considered statistically significant (bilateral).

## Results

### Clinical characteristics of patients

**Figure 1:**
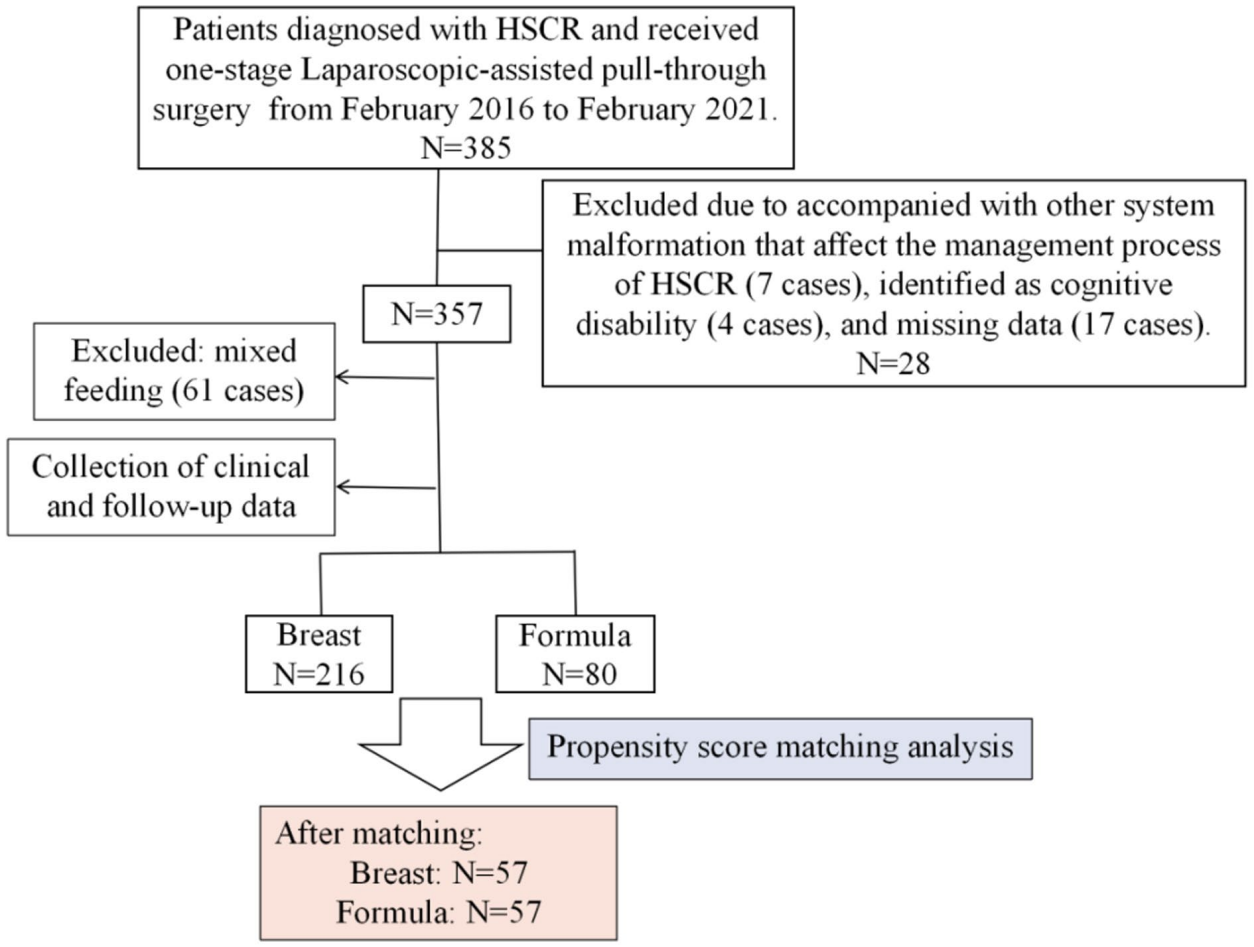
Flow chart of the study population.

The entire number of patients met the in-and exclusion criteria during this time frame was 296 (Figure 1). Clinical data of these patients before and after PSM are presented in Table 1. Of these, 216 cases (73%) were breast-fed and 80 cases (27%) were formula-fed. The following variables were statistically different between breast feeding and formula feeding groups: sex, relationship of caregiver, residence, preoperative HAEC, comorbidity present, preoperative hypoproteinemia, preoperative nutritional status, type of HSCR, surgical time, and age at the last follow-up (*p* < 0.05). However, they were comparable in terms of birth weight, gestational age, educational level of caregiver, insurance type, surgical age, and postoperative time at the last follow-up (*p* > 0.05).

**Table 1:** Clinical data before and after PSM matching in different feeding patterns.

Variables	Before PSM					After PSM				
	Total (*n* = 296)	Breast (*n* = 216)	Formula (*n* = 80)	*P*	SMD	Total (*n* = 114)	Breast (*n* = 57)	Formula (*n* = 57)	*P*	SMD
Sex, *n* (%)				0.022					1.000	
Male	240 (81.08)	182 (84.26)	58 (72.50)		−0.263	90 (78.95)	45 (78.95)	45 (78.95)		0.000
Female	56 (18.92)	34 (15.74)	22 (27.50)		0.263	24 (21.05)	12 (21.05)	12 (21.05)		0.000
Low birth weight (*n*/%)				0.332					0.675	
No	274 (92.57)	198 (91.67)	76 (95.00)		0.153	108 (94.74)	53 (92.98)	55 (96.49)		0.191
Yes	22 (7.43)	18 (8.33)	4 (5.00)		−0.153	6 (5.26)	4 (7.02)	2 (3.51)		−0.191
Premature birth (*n*/%)				0.468					0.714	
No	272 (91.89)	200 (92.59)	72 (90.00)		−0.086	106 (92.98)	54 (94.74)	52 (91.23)		−0.124
Yes	24 (8.11)	16 (7.41)	8 (10.00)		0.086	8 (7.02)	3 (5.26)	5 (8.77)		0.124
Educational level of caregiver (*n*/%)				0.712					0.695	
Secondary and tertiary	179 (60.47)	132 (61.11)	47 (58.75)		−0.048	74 (64.91)	36 (63.16)	38 (66.67)		0.074
Primary and below	117 (39.53)	84 (38.89)	33 (41.25)		0.048	40 (35.09)	21 (36.84)	19 (33.33)		−0.074
Relationship of caregiver (*n*/%)				<0.001					0.826	
Parents	236 (79.73)	187 (86.57)	49 (61.25)		−0.520	87 (76.32)	44 (77.19)	43 (75.44)		−0.041
Others	60 (20.27)	29 (13.43)	31 (38.75)		0.520	27 (23.68)	13 (22.81)	14 (24.56)		0.041
Residence (*n*/%)				0.048					0.695	
Urban	176 (59.46)	121 (56.02)	55 (68.75)		0.275	74 (64.91)	38 (66.67)	36 (63.16)		−0.073
Rural	120 (40.54)	95 (43.98)	25 (31.25)		−0.275	40 (35.09)	19 (33.33)	21 (36.84)		0.073
Insurance type (*n*/%)				0.785					1.000	
Private or self-pay	32 (10.81)	24 (11.11)	8 (10.00)		−0.037	8 (7.02)	4 (7.02)	4 (7.02)		0.000
Public	264 (89.19)	192 (88.89)	72 (90.00)		0.037	106 (92.98)	53 (92.98)	53 (92.98)		0.000
Preoperative HAEC (*n*/%)				<0.001					0.691	
No	234 (79.05)	193 (89.35)	41 (51.25)		−0.762	76 (66.67)	37 (64.91)	39 (68.42)		0.075
Yes	62 (20.95)	23 (10.65)	39 (48.75)		0.762	38 (33.33)	20 (35.09)	18 (31.58)		−0.075
Comorbidity present (*n*/%)				0.005					1.000	
No	265 (89.53)	200 (92.59)	65 (81.25)		−0.291	98 (85.96)	49 (85.96)	49 (85.96)		0.000
Yes	31 (10.47)	16 (7.41)	15 (18.75)		0.291	16 (14.04)	8 (14.04)	8 (14.04)		0.000
Preoperative hypoproteinemia (*n*/%)				0.004					1.000	
No	260 (87.84)	197 (91.20)	63 (78.75)		−0.304	94 (82.46)	47 (82.46)	47 (82.46)		0.000
Yes	36 (12.16)	19 (8.80)	17 (21.25)		0.304	20 (17.54)	10 (17.54)	10 (17.54)		0.000
Preoperative nutritional status (*n*/%)				<0.001					0.570	
Normal	201 (67.91)	164 (75.93)	37 (46.25)		−0.595	68 (59.65)	36 (63.16)	32 (56.14)		−0.141
Risk of malnutrition	62 (20.95)	39 (18.06)	23 (28.75)		0.236	28 (24.56)	14 (24.56)	14 (24.56)		0.000
Malnutrition	33 (11.15)	13 (6.02)	20 (25.00)		0.438	18 (15.79)	7 (12.28)	11 (19.30)		0.178
Surgical age (month, *n*/%)				0.197					0.885	
~ ≤ 1	48 (16.22)	30 (13.89)	18 (22.50)		0.206	28 (24.56)	13 (22.81)	15 (26.32)		0.080
>1 ~ 12	141 (47.64)	104 (48.15)	37 (46.25)		−0.038	44 (38.6)	24 (42.11)	20 (35.09)		−0.147
>12 ~ 36	71 (23.99)	52 (24.07)	19 (23.75)		−0.008	33 (28.95)	16 (28.07)	17 (29.82)		0.038
>36	36 (12.16)	30 (13.89)	6 (7.50)		−0.243	9 (7.89)	4 (7.02)	5 (8.77)		0.062
Type of HSCR (*n*/%)				<0.001					0.635	
S-HSCR	256 (86.49)	203 (93.98)	53 (66.25)		−0.586	92 (80.7)	47 (82.46)	45 (78.95)		−0.086
L-HSCR	40 (13.51)	13 (6.02)	27 (33.75)		0.586	22 (19.3)	10 (17.54)	12 (21.05)		0.086
Surgical time (minute)	142.19 ± 18.11	140.69 ± 17.02	146.24 ± 20.34	0.032	0.273	141.05 ± 17.48	140.30 ± 17.73	141.81 ± 17.34	0.647	0.087
Age at last follow-up (month)	79.08 ± 27.64	80.96 ± 29.79	74.00 ± 20.06	0.022	−0.347	74.71 ± 17.78	74.26 ± 15.59	75.16 ± 19.86	0.788	0.045
Postoperative time (month)	62.69 ± 14.42	63.30 ± 15.20	61.06 ± 12.00	0.188	−0.187	62.04 ± 12.00	62.65 ± 13.04	61.44 ± 10.94	0.592	−0.111

Data are presented as n (%) or mean ± standard deviation and used Chi-square test or Student’s *t* test for analysis.

HSCR, Hirschsprung’s disease; HAEC, Hirschsprung-associated enterocolitis; PSM, propensity score matching; SMD, standardized mean difference.

**Figure 2:**
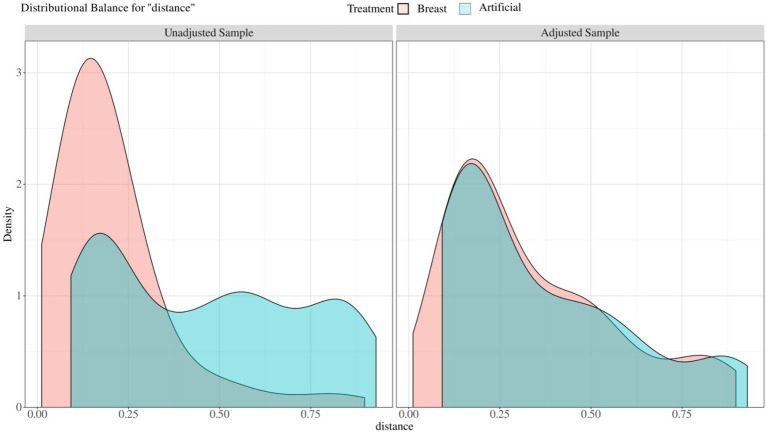
Balance of covariates before and after propensity score matching between different feeding patterns. Unadjusted sample: before matched covariate equalization: Adjust sample: after matched covariate equalization.

Before PSM, some imbalance between the individual propensity score components may be observed (Figure 2). Thus, we made PSM to minimize allocation bias and better represent the associations between feeding patterns and clinical outcomes of HSCR after surgery. After PSM, the SMD values for covariates in the different feeding patterns were all within 0.20, indicating that the potential confounders of the two groups were successfully balanced. After 1:1 PSM, 57 of 216 patients in the breast feeding group (26.39%) were successfully matched to 57 patients of 80 in the formula feeding group (71.25%). The two groups were well balanced in their baseline characteristics after matching (*p* > 0.05 and SMD < 0.20, Table 1; Figure 2).

### Comparison of postoperative outcomes

Table 2 demonstrates the clinical outcomes of HSCR after surgery in breast feeding and formula feeding groups. In the analysis using all patients, which was inevitably subject to the difference in patient background between the groups, formula feeding was significantly associated with a higher rate of postoperative complications within 30 days (mild: 18.75% vs. 14.81%; severe: 17.50% vs. 3.24%, *p* < 0.001), undernutrition (risk of malnutrition: 21.25% vs. 15.28%; malnutrition: 37.50% vs. 6.94%, *p* < 0.001), HAEC (57.50% vs. 17.13%, p < 0.001), and bowel dysfunction (70.0% vs. 44.91%, *p* < 0.001). Furthermore, bowel function score, total HRQOL, three domains of HRQOL (emotional, social, and school functioning) were all significantly higher in the breast feeding group compared with the formula feeding group; 17.24 ± 1.93 vs. 15.57 ± 2.57, 95.92 ± 4.19 vs. 93.66 ± 5.08, 94.26 ± 8.26 vs. 91.49 ± 10.02, 98.65 ± 3.80 vs. 96.10 ± 5 0.98, and 91.68 ± 10.41 vs. 88.75 ± 8.94, respectively (all *p* < 0.05). However, there were no differences in the length of postoperative hospital stay and physical score of HRQOL between two groups.

**Table 2:** Comparison of postoperative outcomes between different feeding patterns.

Variables	Before PSM				After PSM			
	Total (*n* = 296)	Breast (*n* = 216)	Formula (*n* = 80)	*P*	Total (*n* = 114)	Breast (*n* = 57)	Formula (*n* = 57)	*P*
Length of postoperative hospital stay (day)	10.80 ± 2.83	10.61 ± 2.69	11.31 ± 3.14	0.058	10.75 ± 2.30	10.51 ± 1.73	10.98 ± 2.75	0.273
Postoperative complications within 30 days, *n* (%)				<0.001				0.181
No	228 (77.03)	177 (81.94)	51 (63.75)		86 (75.44)	46 (80.70)	40 (70.18)	
Mild	47 (15.88)	32 (14.81)	15 (18.75)		16 (14.04)	8 (14.04)	8 (14.04)	
Severe	21 (7.09)	7 (3.24)	14 (17.50)		12 (10.53)	3 (5.26)	9 (15.79)	
Postoperative HAEC, *n* (%)				<0.001				0.006
No	213 (71.96)	179 (82.87)	34 (42.50)		74 (64.91)	44 (77.19)	30 (52.63)	
Yes	83 (28.04)	37 (17.13)	46 (57.50)		40 (35.09)	13 (22.81)	27 (47.37)	
Postoperative nutritional status, *n* (%)				<0.001				0.023
Normal	201 (67.91)	168 (77.78)	33 (41.25)		72 (63.16)	43 (75.44)	29 (50.88)	
Risk of malnutrition	50 (16.89)	33 (15.28)	17 (21.25)		17 (14.91)	5 (8.77)	12 (21.05)	
Malnutrition	45 (15.20)	15 (6.94)	30 (37.50)		25 (21.93)	9 (15.79)	16 (28.07)	
Bowel function score	16.79 ± 2.25	17.24 ± 1.93	15.57 ± 2.57	<0.001	16.63 ± 2.34	17.14 ± 2.25	16.12 ± 2.33	0.019
Bowel dysfunction, *n* (%)				<0.001				0.018
No	143 (48.31)	119 (55.09)	24 (30.00)		53 (46.90)	33 (57.89)	20 (35.71)	
Yes	153 (51.69)	97 (44.91)	56 (70.00)		60 (53.10)	24 (42.11)	36 (64.29)	
Total HRQOL	95.31 ± 4.55	95.92 ± 4.19	93.66 ± 5.08	<0.001	94.73 ± 4.91	95.01 ± 4.81	94.45 ± 5.04	0.544
Physical	98.85 ± 3.01	99.06 ± 2.82	98.30 ± 3.42	0.053	98.85 ± 3.23	98.55 ± 3.75	99.15 ± 2.61	0.326
Emotional	93.52 ± 8.84	94.26 ± 8.26	91.49 ± 10.02	0.029	93.45 ± 8.55	93.44 ± 8.85	93.46 ± 8.33	0.991
Social	97.96 ± 4.63	98.65 ± 3.80	96.10 ± 5.98	<0.001	97.58 ± 5.13	98.27 ± 3.60	96.89 ± 6.25	0.150
School functioning	90.89 ± 10.11	91.68 ± 10.41	88.75 ± 8.94	0.027	89.05 ± 10.61	89.78 ± 11.57	88.32 ± 9.60	0.465

Data are presented as *n* (%) or mean ± standard deviation and used Chi-square test or Student’s *t* test for analysis.

PSM, Propensity score matching; HAEC, Hirschsprung-associated enterocolitis; HRQOL, health-related quality of life.

The comparison in the propensity-matched patients showed that formula feeding was significantly associated with an increased incidence in postoperative undernutrition (risk of malnutrition: 21.05% vs. 8.77%; malnutrition: 28.07% vs. 15.79%, *p* = 0.023), HAEC (47.37% vs. 22.81%, *p* = 0.006), and bowel dysfunction (64.29% vs. 42.11%, *p* = 0.018).

### Multivariate analysis for postoperative HAEC and bowel dysfunction

Univariate and multivariate logistic regression analyses were undertaken to identify independent factors for postoperative HAEC and bowel dysfunction in the propensity-matched cohort (Tables 3, 4). It should be stated that the effect of feeding patterns on postoperative nutritional status has been analyzed in our previous study (13), so no superfluous description been made here.

**Table 3:** Univariate/multivariate logistic regression analysis of clinical data on the postoperative HAEC (After PSM).

Variables	Univariate				Multivariate			
	*β*	SE	*P*	OR (95%CI)	*β*	SE	*P*	OR (95%CI)
Sex (male)	−0.88	0.55	0.107	0.41 (0.14 ~ 1.21)				
Low birth weight (yes)	−0.08	0.89	0.926	0.92 (0.16 ~ 5.26)				
Premature birth (yes)	0.11	0.76	0.882	1.12 (0.25 ~ 4.95)				
Educational level of caregiver (primary and below)	0.16	0.41	0.692	1.18 (0.53 ~ 2.62)				
Relationship of caregiver (others)	2.01	0.49	<0.001	7.46 (2.86 ~ 19.51)	2.35	0.77	0.002	10.44 (2.33 ~ 46.77)
Residence (rural)	−0.53	0.43	0.214	0.59 (0.26 ~ 1.36)				
Insurance type (private or self-pay)	−0.66	0.74	0.366	0.51 (0.12 ~ 2.18)				
Preoperative HAEC (yes)	2.48	0.47	<0.001	11.90 (4.74 ~ 29.87)	1.58	0.68	0.021	4.85 (1.27 ~ 18.53)
Comorbidity present (yes)	0.72	0.54	0.184	2.06 (0.71 ~ 6.00)				
Preoperative hypoproteinemia (yes)	1.53	0.52	0.003	4.61 (1.66 ~ 12.80)	1.31	0.93	0.161	3.70 (0.59 ~ 23.05)
Preoperative nutritional status*								
Risk of malnutrition	1.68	0.49	<0.001	5.38 (2.04 ~ 14.20)	1.07	0.69	0.120	2.91 (0.76 ~ 11.17)
Malnutrition	2.50	0.61	<0.001	12.13 (3.64 ~ 40.49)	2.94	1.16	0.011	18.91 (1.96 ~ 182.41)
Surgical age* (month)								
>1 ~ 12	−1.36	0.51	0.007	0.26 (0.10 ~ 0.69)	−2.19	0.84	0.009	0.11 (0.02 ~ 0.58)
>12 ~ 36	−1.78	0.59	0.003	0.17 (0.05 ~ 0.54)	−1.71	0.97	0.079	0.18 (0.03 ~ 1.22)
>36	−0.13	0.77	0.871	0.88 (0.20 ~ 3.99)	0.01	1.07	0.990	1.01 (0.13 ~ 8.17)
Type of HSCR (L-HSCR)	1.49	0.50	0.003	4.44 (1.67 ~ 11.84)	3.06	0.98	0.002	21.36 (3.16 ~ 144.44)
Surgical time (minute)	0.02	0.01	0.183	1.02 (0.99 ~ 1.04)				
Feeding method (formula)	1.11	0.41	0.007	3.05 (1.36 ~ 6.83)	1.93	0.70	0.006	6.86 (1.76 ~ 26.79)

*Setting of dummy variables in ordered rank data: surgical age, ~ ≤ 1 month; preoperative nutritional status, normal. HSCR, Hirschsprung’s disease; HAEC, Hirschsprung-associated enterocolitis; *β*, regression coefficient; SE, standard error; OR, odds ratio; CI, confidence interval.

**Table 4:** Univariate/multivariate logistic regression analysis of clinical data on the postoperative bowel dysfunction (After PSM).

Variables	Univariate				Multivariate			
	*β*	SE	*P*	OR (95%CI)	*β*	SE	*P*	OR (95%CI)
Sex (male)	−0.13	0.46	0.771	0.88 (0.36 ~ 2.15)				
Low birth weight (yes)	−1.80	1.11	0.107	0.17 (0.02 ~ 1.47)				
Premature birth (yes)	0.44	0.76	0.565	1.55 (0.35 ~ 6.80)				
Educational level of caregiver (primary and below)	−0.32	0.39	0.420	0.73 (0.34 ~ 1.58)				
Relationship of caregiver (others)	1.74	0.54	0.001	5.67 (1.97 ~ 16.37)	1.03	0.70	0.137	2.81 (0.72 ~ 11.01)
Residence (rural)	−0.63	0.40	0.113	0.53 (0.24 ~ 1.16)				
Insurance type (private or self-pay)	−0.44	0.76	0.565	0.65 (0.15 ~ 2.85)				
Preoperative HAEC (yes)	1.75	0.46	<0.001	5.75 (2.33 ~ 14.22)	1.15	0.58	0.046	3.17 (1.02 ~ 9.87)
Comorbidity present (yes)	2.07	0.78	0.008	7.91 (1.71 ~ 36.68)	2.16	0.88	0.013	8.70 (1.56 ~ 48.44)
Preoperative hypoproteinemia (yes)	0.12	0.49	0.815	1.12 (0.43 ~ 2.96)				
Preoperative nutritional status*								
Risk of malnutrition	1.40	0.49	0.004	4.04 (1.55 ~ 10.49)	1.48	0.58	0.011	4.39 (1.40 ~ 13.74)
Malnutrition	1.73	0.62	0.005	5.65 (1.68 ~ 19.04)	1.63	0.79	0.040	5.10 (1.08 ~ 24.13)
Surgical age* (month)								
>1 ~ 12	−0.01	0.49	0.978	0.99 (0.38 ~ 2.57)				
>12 ~ 36	−0.72	0.52	0.169	0.49 (0.18 ~ 1.36)				
>36	0.41	0.80	0.614	1.50 (0.31 ~ 7.25)				
Type of HSCR (L-HSCR)	2.06	0.66	0.002	7.88 (2.18 ~ 28.48)	1.88	0.78	0.016	6.57 (1.43 ~ 30.28)
Surgical time (minute)	0.02	0.01	0.047	1.02 (1.01 ~ 1.05)	−0.00	0.02	0.760	1.00 (0.97 ~ 1.03)
Feeding method (formula)	0.86	0.38	0.026	2.36 (1.11 ~ 5.00)	1.06	0.51	0.039	2.88 (1.06 ~ 7.83)

*Setting of dummy variables in ordered rank data: surgical age, ~ ≤ 1 month; preoperative nutritional status, normal. HSCR, Hirschsprung’s disease; HAEC, Hirschsprung-associated enterocolitis; β, regression coefficient; SE, standard error; OR, odds ratio; CI, confidence interval.

Significant differences between non-HAEC and HAEC groups were seen in relationship of caregiver, preoperative HAEC, preoperative nutritional status, preoperative hypoproteinemia, type of HSCR, surgical age, and feeding method (all *p* < 0.05, Table 3). These factors were included in the multivariate Logistic regression analysis, which revealed the following independent factors for postoperative HAEC (*p* < 0.05): relationship of caregiver, preoperative HAEC, preoperative nutritional status, preoperative hypoproteinemia, type of HSCR, surgical age, and feeding method (all *p* < 0.05). Significant influenced factors were included in the multivariate Logistic regression analysis, and the results for each category of determinants are listed in Table 3. For postoperative HAEC, significant independent factors were (*p* < 0.05 for all): non-parental caregivers [OR (95% CI) = 10.44 (2.33 ~ 46.77)], preoperative HAEC [OR (95% CI) = 4.85 (1.27 ~ 18.53)], preoperative malnutrition [reference: normal; OR (95% CI) = 18.91 (1.96 ~ 182.41)], L-HSCR [OR (95% CI) = 21.36 (3.16 ~ 144.44)], formula feeding [OR (95% CI) = 6.86 (1.76 ~ 26.79)], and surgical age at >1 ~ 12 month [reference: ≤1 month; OR (95% CI) = 0.11 (0.02 ~ 0.58)].

Similarly, for postoperative bowel dysfunction, the following 7 variables were included in the multivariate analysis: relationship of caregiver, preoperative HAEC, comorbidity present, preoperative nutritional status, type of HSCR, surgical time, and feeding method (all *p* < 0.05, Table 4) and the results showed that preoperative HAEC [OR (95% CI) = 3.17 (1.02 ~ 9.87)], comorbidity present [OR (95% CI) = 8.70 (1.56 ~ 48.44)], preoperative undernutrition [reference: normal; risk of malnutrition: OR (95% CI) = 4.39 (1.40 ~ 13.74); malnutrition: OR (95% CI) = 5.10 (1.08 ~ 24.13)], L-HSCR [OR (95% CI) = 6.57 (1.43 ~ 30.28)], and formula feeding [OR (95% CI) = 2.88 (1.06 ~ 7.83)] were independent factors for postoperative bowel dysfunction (all *p* < 0.05).

To explore whether the effect of feeding patterns on postoperative outcomes varies across clinically relevant subgroups, we performed further subgroup analysis on this cohort. It should be noted that due to the influence of the sample size after matching, we found that some variables showed extreme value effects in the subgroup analysis; so we only selected the indicators of interest for stratification based on clinical practice. For the subgroup analysis of postoperative HAEC, among the patients surgical aged >1 and ≤12 months, the use of formula could significantly increase the incidence of postoperative HAEC (Table 5). In terms of postoperative bowel dysfunction, subgroup analyses revealed pronounced adverse impact in patients with S-HSCR (Table 5).

**Table 5:** Subgroups analysis for feeding patterns on postoperative outcomes.

Variables	*n* (%)	OR (95%CI)	*P*	*P* for interaction
Postoperative HAEC				
All patients	114 (100.00)	3.05 (1.36 ~ 6.83)	0.007	
Residence				0.335
Urban	74 (64.91)	7.28 (1.86 ~ 28.45)	0.004	
Rural	40 (35.09)	23.80 (1.45 ~ 390.19)	0.026	
Surgical age (month)				0.701
~ ≤ 1	28 (24.56)	10.66 (0.93 ~ 121.91)	0.057	
>1 ~ 12	44 (38.60)	6.32 (1.22 ~ 32.84)	0.028	
>12 ~ 36	33 (28.95)	1.88 (0.14 ~ 25.32)	0.636	
>36	9 (7.89)	1.00 (0.03 ~ 29.81)	1.000	
Postoperative bowel dysfunction				
All patients	114 (100.00)	2.36 (1.11 ~ 5.00)	0.026	
Relationship of caregiver				0.827
Parents	87 (76.32)	2.51 (0.96 ~ 6.53)	0.059	
Others	27 (23.68)	0.78 (0.03 ~ 24.00)	0.887	
Preoperative HAEC				0.926
No	76 (66.67)	2.72 (0.97 ~ 7.62)	0.057	
Yes	38 (33.33)	2.12 (0.46 ~ 9.78)	0.335	
Type of HSCR				0.106
S-HSCR	92 (80.70)	3.28 (1.31 ~ 8.21)	0.011	
L-HSCR	22 (19.30)	0.53 (0.03 ~ 8.41)	0.654	

OR, odds ratio; CI, confidence interval; HSCR, Hirschsprung’s disease; HAEC, Hirschsprung-associated enterocolitis.

## Discussion

HSCR patients who receive surgery at the appropriate time tend to demonstrate good outcomes and clinical improvement. However, about one-third of patients still face a series of postoperative problems. It’s known that nutritional needs in early life are critical for children and directly affect their health, growth and development (15). As an important therapeutic measure to improve the clinical outcome of patients, breast feeding has been paid more and more attention by clinicians (16, 17). Several reports have reported that the appropriate feeding pattern is of great significance in maintaining the nutritional balance, promoting wound healing, improving immunity and reducing complications (18, 19). However, the benefit of breast feeding on clinical outcomes of HSCR after surgery remains unknown.

In this study, we applied propensity scoring with full matching methodology to a single-institutional cohort of 296 patients with HSCR to estimate associations between feeding patterns and surgical outcomes, with a view to providing reference and guidance for clinical practice. It found that the baseline characteristics of patients between the groups were not comparable in the entire cohort, the following factors account for the statistical differences: sex, relationship of caregiver, residence, preoperative HAEC, comorbidity present, preoperative hypoproteinemia and nutritional status, type of HSCR, surgical time, and age at last follow-up. We minimized these allocation bias by using PSM method to adjust for significant differences in baseline characteristics. After adjustment of propensity score, other covariates, except feeding patterns, can be balanced and comparable so that non-random grouping data can be used to study the relationship between trial factors and outcomes, and obtain more reliable research results (20). The results obtained show that formula feeding is associated with the adverse outcomes for HSCR after surgery, including postoperative undernutrition, bowel dysfunction, and HAEC. Furthermore, we found that formula feeding was independent risk factors for both postoperative bowel dysfunction and HAEC. Taken together, these data support the superiority of breast feeding for postoperative outcomes in patients with HSCR.

Early life nutrition is fundamental to children’s growth and development. Breast feeding can not only meet the nutritional needs of infant growth and development, but also promote infant organ development and functional maturity. In addition, breast milk is enriched with a diverse array of immunoactive substances, reasonable breast feeding can reduce infant morbidity (21). Over the past few decades, there has been increasing evidence of the health benefits of breast feeding for both mothers and infants, and the benefits for children can be extended into adulthood. A global survey of feeding patterns under 6 months of age in 2021 shows that 44% of infants are exclusively breastfed, with a lower proportion particularly in the Low-and Middle-Income Countries (22). Previous survey show that the overall rate of breast feeding in our country (urban: 48.8%; rural: 48.4%) is significantly lower than the world average level, which also shows a large regional difference (23). In this cohort, the breast feeding rate in patients with HSCR was 60.6% (216/357), significantly higher than that reported in previous studies (8). This may be due to the Chinese government’s active promotion of breast feeding in the past decade and vigorously calls for construction of baby-friendly hospitals and breast feeding week activities (24). With the increasing awareness of breast feeding, mothers tend to use breast milk as the preferred food for infant feeding. This study has enriched the benefits of breast feeding in patients with HSCR, and its improvement for clinical outcomes is noteworthy. It should be stated that our previous report have confirmed and explained that breast feeding helps to improve the nutritional status of HSCR after surgery (13), so we focus here on discussing its effects on postoperative HAEC and bowel function.

It has been shown that the occurrence of postoperative HAEC is closely related to the complex interaction of intestinal microbial plexus, intestinal mucosal barrier and intestinal immune system, which has prompted extensive global research into intestinal ecology (25). These studies revealed that the reduction in the diversity of intestinal microbial plexus, coupled with disruption of intestinal mucosal barrier, significantly contributes to the development of postoperative HAEC (26, 27). Furthermore, Secretory immunoglobulin A (sIgA), one of the major immunoglobulins in the gastrointestinal tract that mainly derived from breast milk during infancy, has been found to be at low levels in patients with HAEC (28, 29). By fostering a richer intestinal microbial plexus, safeguarding the integrity of the intestinal mucosal barrier, and enhancing the body’s innate immune and anti-inflammatory capabilities, breast feeding can effectively mitigate the risk of postoperative HAEC in patients with HSCR (9, 30). To date, several risk factors for postoperative HAEC have been identified, eg. L-HSCR, preoperative undernutrition and HAEC, and surgical age (11, 31). Our results are basically consistent with these previous findings. Furthermore, residence and surgical age may be associated with the occurrence of postoperative HAEC. We did subgroup analysis to rule out the confounding factors that might affect the results. However, based on the results of this study, residence and surgical age at >1 ~ 12 months have an effect on the outcomes. Due to the limitation of the sample size, the effect of confounding factors needs to be further verified in future study. With reference to these risk factors, targeted clinical management may be beneficial in reducing the incidence of postoperative HAEC. Breast feeding, a patient-dependent modifiable factor, is worth advocating for pregnant women, especially if prenatal screening is found to be a high-risk population for HSCR.

Interestingly, the results showed that feeding patterns were associated with postoperative defecation function. Bowel dysfunction, mainly including constipation and fecal incontinence, is a concern worthy of attention for patients with anorectal diseases after surgery. Due to the change in anatomic structure, patients with HSCR still have imbalance of gut microbiota homeostasis and intestinal immunity even after radical surgery, leading to bowel dysfunction. As described above, breast feeding is beneficial for maintaining gut microbiota homeostasis and enhancing intestinal immunity. It has shown that breast feeding can mitigate the risk of constipation (18), whereas the incidence of constipation-related symptoms escalates in parallel with declining vitamin-D levels, notably among individuals with gastrointestinal involvement extending to the colorectum (32). Paradoxically, breastfed infants exhibit lower vitamin-D concentrations, suggesting a complex interplay that necessitates further investigation into the underlying mechanism by which breast feeding affects constipation (33). Furthermore, it has found that low levels of sIgA were a risk factor for postoperative fecal incontinence in patients with HSCR (28), whereas breast milk uniquely contains a specific sIgA variant that can enhance mucosal defense in infants (34). Of note, our previous study has shown that the occurrence of HAEC was associated with worse postoperative bowel function (11). In the subgroup analysis, we also included relationship of caregiver, preoperative HAEC, and type of HSCR to identify potential effect modifiers and offer more tailored clinical insights. And the result showed that relationship of caregiver and preoperative HAEC had no effect on the outcomes, but the S-HSCR had an influence on the relationship between formula feeding and postoperative bowel dysfunction. Taken together with our analysis above, it suggests that breast feeding may improve bowel function by reducing the occurrence of HAEC, but the exact mechanism needs to be further explored.

Enteral nutrition stands as the sole avenue for nutrient intake during infancy, underscoring its paramount importance. Our study underscores the profound impact of breast feeding on postoperative clinical outcomes in patients with HSCR. This simple shift in feeding practices, feasible in hospitals and homes, has profound societal, familial, and individual benefits (8). It promotes social well-being, reduces healthcare burdens, strengthens family bonds, and improves clinical outcomes for patients with HSCR, laying a strong foundation for their growth and future health. Thus, advocating breast feeding as the optimal nutrition strategy for these patients offers a powerful means to positively transform lives across communities.

## Limitation

Several limitations of this study warrant acknowledgment. First, this study is limited by its retrospective design, and non-standardized data collection may have obscured other significant factors or potential confounders (i.e.wealth index and maternal dietary habits). Second, 43 cases receiving staged procedure were not included in analysis due to the small sample size and inconsistent surgical strategies. Thus, the advantages of breast feeding in these patients deserve further study. Finally, mixed feeding was not examined due to the indeterminate ratio of breast milk to formula. Further research, especially large-scale and multi-center prospective studies with comprehensive data collection, is necessary to validate the influences of feeding practices on clinical outcomes for HSCR after surgery.

## Conclusion

To the best of our knowledge, this is the first study to establish a link between feeding patterns and clinical outcomes of HSCR after surgery. Although only a few variables were analyzed, we successfully identified that breast feeding is associated with the better postoperative outcomes for these patients compared with formula feeding, including postoperative nutritional status, bowel function, and the occurrence of HAEC, indicating the superiority and importance of breast feeding. In conclusion, the findings of our study not only provides a theoretical basis for the promotion of breast feeding in HSCR patients, but also helps physicians fully communicate complications with family members.

## Data Availability

The original contributions presented in the study are included in the article/supplementary material, further inquiries can be directed to the corresponding author/s.
